# Boycotting the Germans

**Published:** 1918-09

**Authors:** 


### TEXAS MEDICAL JOURNAL

DR. F. E. DANIEL,
Founder.

Established July,
1885

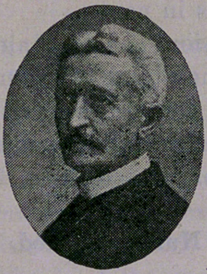

MRS. F. E. DANIEL,
Publisher and Managing
Editor.

VOL. XXXIV No. 3
AUSTIN,
SEPTEMBER, 1918.

PUBLISHED MONTHLY.
Subscription:
$1.50

The publisher is not responsible for views of contributors.

ASSISTANT EDITORS:
DR. JOHN S. TURNER, Dallas, Texas. DR. JOE BECTON, Greenville, Texas.
DR. E. B. PARSONS, Palestine, Texas. DR. M. F. BLEDSOE, Port Arthur.
DR. A. P. HOWARD, Houston, Texas. DR. J. H. FLORENCE, Dallas, Texas.
DR. J. H. REUSS, Cuero, Texas.

*"All of good the past hath had,
Remains to make our own time glad."*—WHITTIER.

### EDITORIAL.

ARMY
MEDICAL
SEP 2 3 1947
LIBRARY

**Boycotting the Germans.**

The president of the American Medical Association, Dr. Arthur Dean Bevin, of Chicago, in his installation speech, declared in favor of boycotting German scientists after the war until their government purges itself of all guilt. That is right. Not only German scientists, but German educators, and Germans of every other class should be boycotted until the country atones for its misdeeds. German business should be cut out of all foreign countries that are not in direct sympathy with the German methods, and those so-called neutral countries that continue any kind of relations with Germany should be placed in the same class with Germany and boycotted.

The boycott should not extend to Germany alone but the German citizen in this country, who does not at once become naturalized and prove by certain tests his complete allegiance to the country should be deported, and no citizen of Germany should be allowed in this country during the life time of any one engaged in this war. America must be Americanized when this war is over and the only way to do it is to be sure that those who enjoy the advantages of citizenship should be citizens at heart as well as in name.

The medical profession can get along without Germany. It is doing so nicely now and can continue to do so forever if need be. Certainly German scientists should be let alone until the country has made full atonement for what it, largely through its activities of its scientists, has done.

